# Intercalation of p-Aminopyridine and p-Ethylenediamine Molecules into Orthorhombic In_1.2_Ga_0.8_S_3_ Single Crystals

**DOI:** 10.3390/ma16062368

**Published:** 2023-03-15

**Authors:** Aysel B. Rahimli, Imamaddin R. Amiraslanov, Zakir A. Jahangirli, Naila H. Aliyeva, Pascal Boulet, Marie-Christine Record, Ziya S. Aliev

**Affiliations:** 1 Institute of Physics, Ministry of Science and Education of Azerbaijan, AZ1143 Baku, Azerbaijan; 2 Nanoresearch Laboratory, Baku State University, AZ1148 Baku, Azerbaijan; 3 Chemical Technologies Department, Faculty of Metallurgy and Materials Science, Azerbaijan Technical University, AZ1073 Baku, Azerbaijan; 4 CNRS MADIREL Laboratory, Faculty of Sciences, Aix-Marseille University, 13013 Marseille, France; 5 CNRS IM2NP Laboratory, Faculty of Sciences, Aix-Marseille University, 13013 Marseille, France

**Keywords:** gallium indium sulfide, single crystal, intercalation compounds, X-ray powder diffraction, Rietveld refinement, Raman scattering, DFT, charge density topology, band structure

## Abstract

A single crystalline layered semiconductor In_1.2_Ga_0.8_S_3_ phase was grown, and by intercalating p-aminopyridine (NH_2_-C_5_H_4_N or p-AP) molecules into this crystal, a new intercalation compound, In_1.2_Ga_0.8_S_3_·0.5(NH_2_-C_5_H_4_N), was synthesized. Further, by substituting p-AP molecules with p-ethylenediamine (NH_2_-CH_2_-CH_2_-NH_2_ or p-EDA) in this intercalation compound, another new intercalated compound—In_1.2_Ga_0.8_S_3_·0.5(NH_2_-CH_2_-CH_2_-NH_2_) was synthesized. It was found that the single crystallinity of the initial In_1.2_Ga_0.8_S_3_ samples was retained after their intercalation despite a strong deterioration in quality. The thermal peculiarities of both the intercalation and deintercalation of the title crystal were determined. Furthermore, the unit cell parameters of the intercalation compounds were determined from X-ray diffraction data (XRD). It was found that increasing the *c* parameter corresponded to the dimension of the intercalated molecule. In addition to the intercalation phases’ experimental characterization, the lattice dynamical properties and the electronic and bonding features of the stoichiometric GaInS_3_ were calculated using the Density Functional Theory within the Generalized Gradient Approximations (DFT-GGA). Nine Raman-active modes were observed and identified for this compound. The electronic gap was found to be an indirect one and the topological analysis of the electron density revealed that the interlayer bonding is rather weak, thus enabling the intercalation of organic molecules.

## 1. Introduction

In recent years, much more interest has been focused on layered van der Waals (vdW) materials due to their unique structural and electronic features. Layered materials can be either single-element crystals, such as graphite, black phosphorus, black arsenic, antimony, or bismuth, or inorganic compounds, such as transition metal chalcogenides, some oxides, silicates, hydroxides, etc. Layered vdW materials usually exhibit strong in-plane covalent bonding while weak van der Waals interactions between the neighboring layers are in play. Most of these materials exhibit a wide range of electronic properties, from dielectric, semiconducting, metallic, and superconducting, to topologically insulating [1,2,3,4,5,6,7,8,9,10].

Intercalating transition metal dichalcogenides with various ions leads to the appearance of curious physical phenomena such as charge density waves, two-dimensional superconductivity, etc. [11,12]. Some organic molecules may also intercalate into the vdW gap of the layered compounds. This kind of intercalation process has been of great interest in recent years from the viewpoint of tuning the transport and optical properties [12,13]. Intercalation phases with organic molecules are often called inorganic-organic hybrid compounds when they are bonded covalently and become new semiconductors with tunable electronic properties. 

Intercalation in some transition metal dichalcogenides has been successfully achieved by chemical transport reactions and electrochemical, ion exchange, and redox methods, in which the embedded substances can be atoms, ions, or molecules [11,12,13,14,15,16,17,18,19,20,21,22,23,24]. In general, the intercalation of various ions or organic molecules into the vdW gap of layered inorganic materials is one of the effective design methods for creating hybrid materials with novel physical properties [25,26].

Depending on the growing conditions, several layered hexagonal In_1+x_Ga_1-x_S_3_ phases crystallize in the In-Ga-S system. The crystal structure of all these phases has already been reported in our previous works [27,28,29,30]. In addition to the hexagonal phases, the orthorhombic phase also crystallizes in the In-Ga-S system [31]. This phase also has a layered structure where indium atoms are in octahedra whereas the gallium atoms are coordinated in tetrahedra.

Previously, we grew InGaS_3_ single crystals and then synthesized their intercalates with p-AP (NH_2_-C_5_H_4_N). X-ray studies revealed that the high-quality matrix single crystals became highly dispersed after the intercalation process, despite the geometric shapes of the crystals remaining stable. Therefore, there were certain difficulties in determining the unit cell parameters of the intercalate. An orthorhombic cell was chosen for the intercalate in previously published works [32,33,34] but the structure has not yet been solved. In addition, the crystal structure refinement of the matrix crystal by the Rietveld method was presented in [34]. It was found that 20% of the tetrahedral positions were occupied by indium atoms (20% In + 80% Ga) and the real composition of the crystals corresponded to the formula In_1.2_Ga_0.8_S_3_. However, due to the partial substitution of the In atoms for Ga, this phase may have had a solid solution region.

Here, we present the results of the X-ray and thermal analysis of In_1.2_Ga_0.8_S_3_ with p-AP and p-EDA (NH_2_-CH_2_-CH_2_-NH_2_) molecules as intercalates. Moreover, this paper reports ab initio calculations of the lattice dynamical properties, and the investigation of the electronic structure and charge density topology of the stoichiometric GaInS_3_, revealing the feasibility of intercalating the molecules within the interlayer space.

## 2. Materials and Methods

### 2.1. Synthesis and Single Crystal Growth of Orthorhombic In_1.2_Ga_0.8_S_3_ Phase

Gallium (99.999 mass%), indium (99.99 mass%), and sulfur (99.999 mass%) purchased from Alfa Aesar were used for the synthesis of the In_1.2_Ga_0.8_S_3_ phase. The synthesis was carried out in an evacuated (under 10^−3^ Pa) quartz ampoule by melting a stoichiometric mixture of the initial elements. An ampoule was heated up to 1050 °C and then slowly cooled down to room temperature for 24 h. The chemical vapor transport (CVT) method was used to grow single crystals of the In_1.2_Ga_0.8_S_3_ phase. In this method, an approximately 1.2 g polycrystalline sample was placed in a quartz ampoule together with elemental iodine whose mass was calculated as 4 mg/cm^3^ regarding the ampoule volume. A loaded ampoule was sealed under 10^−3^ Pa and then placed in the middle of a horizontal two-zone furnace. The temperature conditions for growing a single crystal were T_1_ = 700 °C and T_2_ = 600 °C. As a result, high-quality single crystals of this phase were achieved.

### 2.2. Synthesis of Intercalation Compounds

The synthesis of the intercalation compound was performed in an evacuated quartz ampoule by direct interaction of orthorhombic In_1.2_Ga_0.8_S_3_ single crystals and p-AP. The mixture was kept above the melting point of p-AP (T_m_ = 158 °C) at a temperature of 180–200 °C for three days. The excess of p-AP was washed from the obtained intercalated crystals with ethyl alcohol. The intercalate compound In_1.2_Ga_0.8_S_3_ with p-EDA molecules was obtained by replacing the p-AP molecules with p-EDA molecules in the In_1.2_Ga_0.8_S_3_·0.5(p-AP) intercalate. For this purpose, the intercalate crystals and p-EDA molecules were placed in a glass ampoule. Then, the ampoule was evacuated, heated to 200 °C, and kept at this temperature for three days.

### 2.3. DTA and TG Analysis

DTA and TG analyses of the samples were performed on a Netzsch STA 449 F3 system with platinum–rhodium thermocouples under an argon gas atmosphere from room temperature up to ~450 K with a heating rate of 15 K/min.

### 2.4. X-ray Diffraction Characterization

XRD analyses of the polycrystalline samples were carried out on BRUKER D2-PHASER and D8-ADVANCE diffractometers (Cu*K*_α_; 5° ≤ 2θ ≤ 100°), from room temperature up to 420 °C, using the EVA and Topas 4.2 software packages supplied together with the diffractometer.

### 2.5. Raman Characterization

Raman scattering studies were performed with the aid of a confocal micro-spectrometer Nanofinder 30 (Tokyo Instruments, Tokyo, Japan). A second harmonic (532 nm) of an Nd:YAG laser with a maximum output power of 10 mW was used as an excitation source, and the cross-sectional beam diameter was 4 μm. A CCD camera cooled down to –70 °C was used for Raman signal detection. The spectra were taken in back-scattering geometry at room temperature.

## 3. Computational Details

### 3.1. Dynamical Properties

The calculations of dynamical properties for the stoichiometric GaInS_3_ phase were performed using the Density Functional Perturbation Theory (DFPT) [35,36,37,38] based on the pseudopotential method as implemented in the ABINIT code [39]. Hartwigsen–Goedecker–Hutter norm-conserving pseudopotentials [40] and the generalized gradient approximation (GGA) exchange-correlation functional of Perdew, Burke, and Ernzerhof (PBE) [41] were used in phonon spectra calculations. A plane wave basis set with a cutoff of 80 Ry was used for the expansion of the electronic wavefunctions, which provided good convergence of the total energy. The integration over the Brillouin zone was solved using the special approximation of k-points on a 4 × 4 × 4 grid provided by the Monkhorst–Pack method [42].

The lattice parameters and the equilibrium atomic positions in the unit cell were determined by the minimization of Hellmann–Feynman forces using the Broyden–Fletcher–Goldfarb–Shanno (BFGS) method and the experimental structure as a starting point. The process of minimization continued until the force moduli became less than 10^−4^ eVÅ^−1^. Initially, the dependence of the convergence of the total energy and the Hellmann–Feynman forces on both the Monkhorst–Pack mesh and the energy cutoff of the plane waves was tested considering the optimal computational cost-to-accuracy ratio.

### 3.2. Electronic Properties and Charge Density Analysis

The PBE exchange-correlation function combined with the DFT-D3(BJ) approach of Grimme et al. [43,44] was used. The projector augmented wave pseudopotentials as implemented in the VASP code [45,46,47] were used. A cutoff energy of 30 eV and a Monkhorst–Pack grid of 13 × 7 × 3 k-points were employed. The structure was optimized with the precision thresholds of 10^−5^ eV and 10^−3^ eVÅ^−1^ for the energy and atomic forces, respectively. For the topological analysis of the charge density, a very fine grid of over 72 × 10^6^ points was used. The topological analysis was performed with the Critic2 program [48,49] that implements the Bader quantum theory of atoms in molecules [50].

## 4. Results and Discussion

### 4.1. Crystal Structure of the Orthorhombic Phase In_1.2_Ga_0.8_S_3_

**Figure 1:**
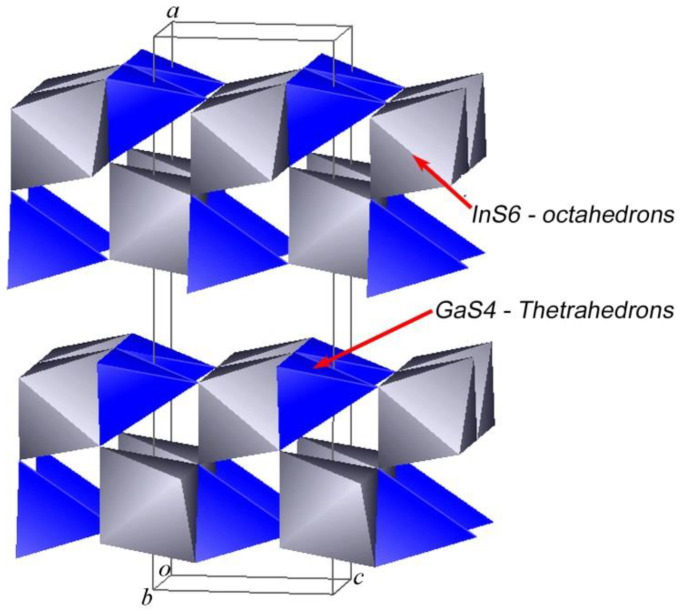
Crystal structure of the orthorhombic In_1.2_Ga_0.8_S_3_ phase.

The crystal structure of the layered orthorhombic In_1.2_Ga_0.8_S_3_ is described in [34] (Person’s card number 1961818 and JCPDS card number 04-026-4498 [51]). As we know, the structure of most layered chalcogenides is built up by a close-packing principle in a hexagonal system. The building blocks of such structures are two-dimensionally infinite packages consisting of several alternating A-K-...-A-type anion-cation layers. A three-dimensional structure is formed by the repetition of such blocks along the “*c*” axis. In this type of structure, the bonds between adjacent packets are the van der Waals type formed by the termination anions of each block. In the case of orthorhombic In_1.2_Ga_0.8_S_3_ crystals, each building package consists of five such alternating layers: S-(Ga, In)-S-(Ga, In)-S. An interesting fact here is that, unlike most layered crystals, the inter-package gaps are characterized by a complex zigzag architecture (Figure 1). In the structure of the orthorhombic phase, the ratio of tetrahedrons and octahedrons is equal.

### 4.2. Intercalation of the p-Aminopyridine into the In_1.2_Ga_0.8_S_3_

**Figure 2:**
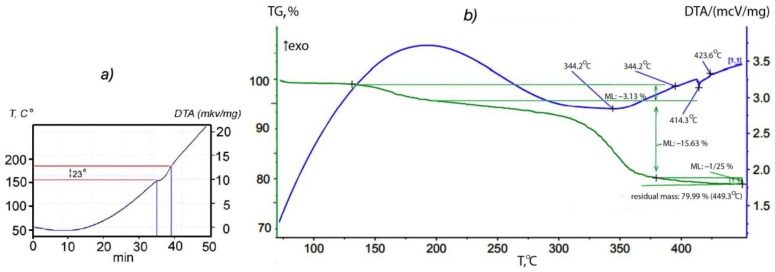
(**a**) Thermogram of a mixture consisting of the crystalline powders of In_1.2_Ga_0.8_S_3_ and p-AP. (**b**) DTA and TG spectrum of the In_1.2_Ga_0.8_S_3_·0.5(p-AP).

The synthesis procedure for the p-AP intercalated orthorhombic In_1.2_Ga_0.8_S_3_ is described in detail in Section 2.2. To determine the temperature of the onset of intercalation, a DTA thermogram of a mixture consisting of crystalline powders of In_1.2_Ga_0.8_S_3_ and p-AP was taken while heated up to 250 °C (Figure 2a). The thermogram of the intercalation process shows only one endothermic effect at 158 °C corresponding to the melting point of crystalline p-AP. The absence of additional thermal effects means that melting and intercalation occur simultaneously. Identification of the chemical composition and deintercalation process was examined further using thermogravimetric (TG) analysis. The DTA spectrum shows clearly exo- and endothermic effects at 140 °C and 344.2 °C, respectively (Figure 2b). The weight loss was ~3.13% and ~15.63% for the first and second effects, respectively. Based on the relatively low temperature and small weight change, it can be assumed that the first effect corresponds to the removal of weakly bounded p-AP molecules whereas the second peak refers to the removal of the main intercalated portion of p-AP. Using the weight loss associated with the second peak and considering the formula unit of the matrix crystals (In_1.2_Ga_0.8_S_3_), the chemical formula of the intercalation compound was found to be In_1.2_Ga_0.8_S_3_·0.5(p-AP).

**Figure 3:**
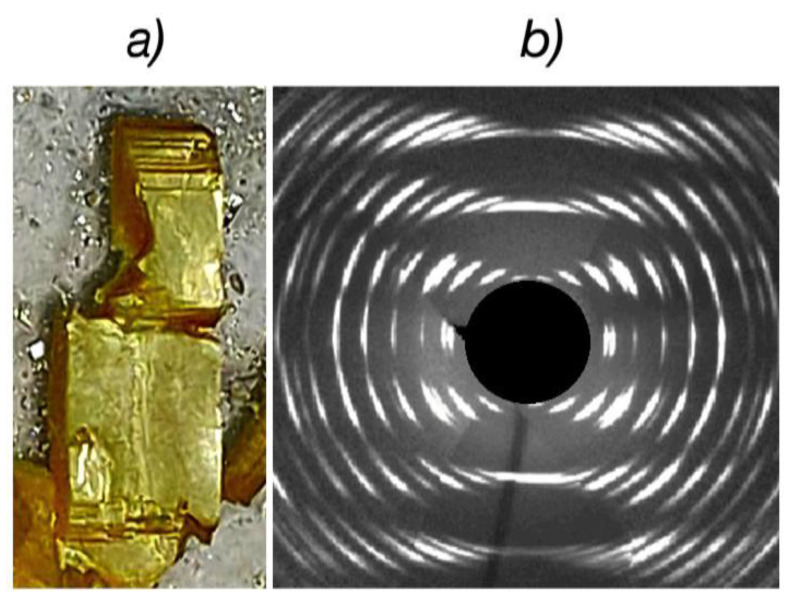
Crystal image (**a**) and XRD pattern (**b**) of the intercalated In_1.2_Ga_0.8_S_3_·0.5(p-AP) crystals.

Figure 3a shows one of the intercalated crystals with well-defined geometric shapes whereas Figure 3b shows its diffraction image. One can see that although single crystals retain their geometric shapes during the intercalation process, the crystallinity is significantly impaired. Despite the strongly diffuse shape of diffraction spots, they still retain their individuality. A significant difference in the distribution of these spots in the horizontal and vertical directions also indicates that the intercalated crystals retain single crystallinity, despite their poor quality. Such a deterioration process makes the determination of the unit cell parameters quite difficult.

**Figure 4:**
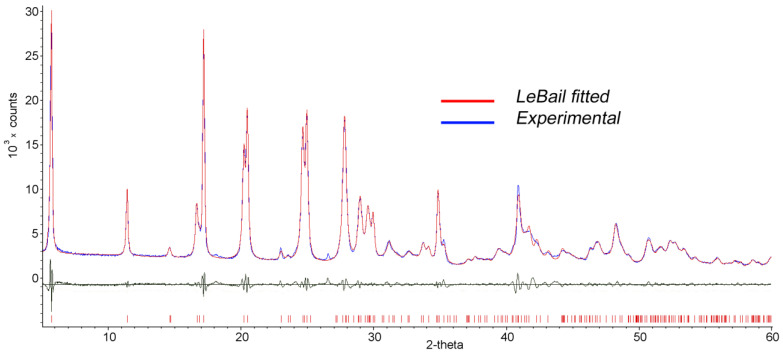
LeBail fitted (red line) and experimental (blue line) diffraction patterns of the In_1.2_Ga_0.8_S_3_·0.5(p-AP) intercalation compound.

In our previous works [32,33], the monoclinic unit cell with the following parameters *c* = 31.12(4), *a* = 3.764(5), *b* = 6.135(7) Å, γ = 90°, and sp. gr. *B*112 was determined for the InGaS_3_+p-AP intercalate. However, these parameters were found to be unsatisfactory for indexing the diffraction pattern of the In_1.2_Ga_0.8_S_3_·0.5(p-AP) compound (Figure 4, blue line) since some diffraction lines remained unindexed. Moreover, attempts to choose an orthorhombic or monoclinic space group corresponding to the model structure have not yielded results due to strong deterioration in the single crystallinity of intercalated crystals. Therefore, in this work, we tried to determine the correct characteristics of the unit cell using the powder diffraction data of the intercalation compound. The best solution, which agreed well with the diffraction image, was a triclinic unit cell with the following characteristics, refined by the LeBail method (Figure 4, red line): Sp. gr. *P*1, *a* = 15.8933(7), *b* = 3.7934(2), *c* = 6.1544(5) (Å), α = 92.076(5), β = 100.750(6), and γ = 81.170(6).

**Figure 5:**
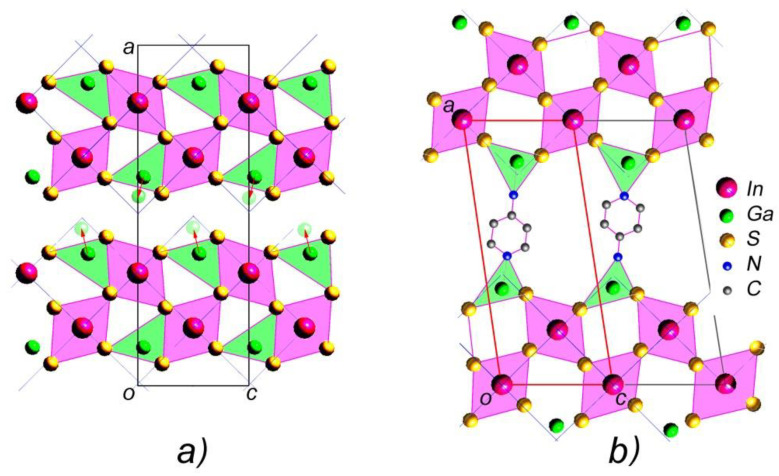
(**a**) The projection of the In_1.2_Ga_0.8_S_3_ crystals and (**b**) the model structure of intercalated In_1.2_Ga_0.8_S_3_·0.5(p-AP).

The lattice parameters *b* and *c* characterize the sizes of blocks of the In_1.2_Ga_0.8_S_3_ crystal structure (Figure 1). As can be seen, their values are very close to the corresponding lattice parameters of the intercalated crystals, meaning that after the intercalation process, no strong changes occur in the geometric dimensions of the blocks. In this case, the *a* parameter determines the rule for the mutual arrangement of neighboring blocks. The intercalation of p-AP molecules between blocks should significantly increase this parameter, which we observed. On the other hand, we know that the deintercalation temperature of the p-AP molecule is 345 °C (Figure 2b), which is quite high for a molecule sharing weak vdW bonds with the host structure. In addition, it is well known that the thermal decomposition of a complex compound usually breaks covalent bonds between an organic molecule and metal atoms at about ≥300 °C [52,53]. Therefore, it can be assumed that the bond of the p-AP molecule with the inorganic matrix is a covalent one. A detailed examination of the structural blocks of In_1.2_Ga_0.8_S_3_ allows us to conclude that such a bond can be formed if the Ga atoms move to a new position, as shown in Figure 5a (red arrows). In this case, the nitrogen atoms of p-AP molecules can form covalent bonds with gallium atoms and connect the blocks into a single framework structure. However, all our attempts to determine the structure of the intercalation compound were unsuccessful due to the low quality of the crystals.

Thus, considering (i) the value of the unit cell parameters, (ii) the displacement of Ga atoms to new positions, (iii) the alternation of blocks in the c direction, and (iv) the formation of covalent bonds between the nitrogen atoms of p-AP with the gallium atoms of the blocks, it is possible to suggest a model structure shown in Figure 5b for intercalated crystals. The small value of parameter *b* (3.793 Å) indicates that the hexagonal ring of p-aminopyridine should be located almost parallel to the *ac* plane.

### 4.3. Deintercalation of In_1.2_Ga_0.8_S_3_·0.5(p-AP) Compound

**Figure 6:**
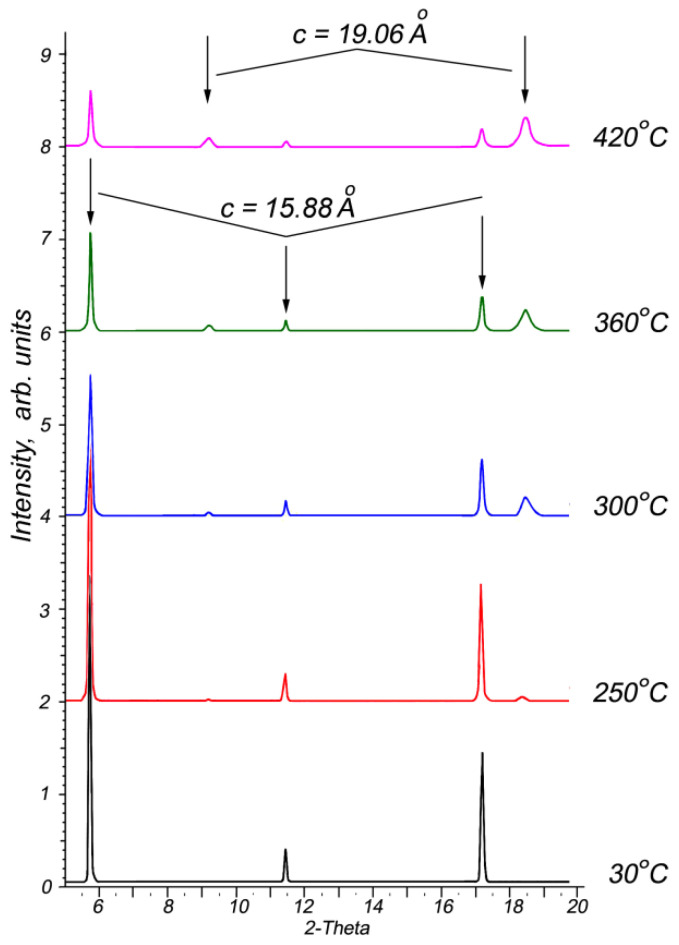
High-temperature X-ray diffraction patterns of In_1.2_Ga_0.8_S_3_·0.5(NH_2_-C_5_H_4_N) crystals in the temperature range of 30–420 °C.

The intercalation↔deintercalation reversibility is a typical property for most intercalation compounds. Therefore, studying the thermal decomposition process to determine the nature of the deintercalation and restoration of the matrix crystals is essential. Thermogravimetric analysis results (Figure 2b) confirm that the intercalated p-AP molecules are completely removed from the intercalation compound upon heating up to 350 °C. Further, the In_1.2_Ga_0.8_S_3_·0.5(p-AP) crystals were studied by high-temperature XRD from room temperature up to 420 °C to follow structural changes. One can see from Figure 6 that the intercalation compound is stable up to 250 °C. However, the weak diffraction peaks that appear at 2θ = ~9.2° and ~18.6° after 250 °C can be referred to In_1.2_Ga_0.8_S_3_ matrix crystals. Upon heating up to 420 °C, the intensities of these lines gradually increase (Figure 6), while the intensities of other peaks, which belong to the intercalation compound, significantly decrease but do not disappear completely.

The XRD pattern at 420 °C clearly shows diffraction peaks related to the intercalated compound, despite the DTA thermogram confirming that the p-AP molecules were completely removed. In other words, the In_1.2_Ga_0.8_S_3_ matrix crystals are found to be not completely restored after the deintercalation process. On the other hand, the appearance at ~250 °C of peaks corresponding to the matrix crystal confirms that the initial state of the crystals is mostly restored.

The deintercalation of the main part of p-AP from the intercalated compound occurs at around 345 °C according to the thermogram (Figure 2b). However, it is a rather high temperature because the decomposition of many complex compounds containing covalent or ionic bonds occurs around this temperature. This confirms that in the In_1.2_Ga_0.8_S_3_·0.5(p-AP) crystals, the p-AP molecules form stronger bonds with matrix crystal atoms than van der Waals or hydrogen bonds.

### 4.4. Raman Spectra of Orthorhombic In_1.2_Ga_0.8_S_3_ and In_1.2_Ga_0.8_S_3_·0.5(p-AP) Compound

**Figure 7:**
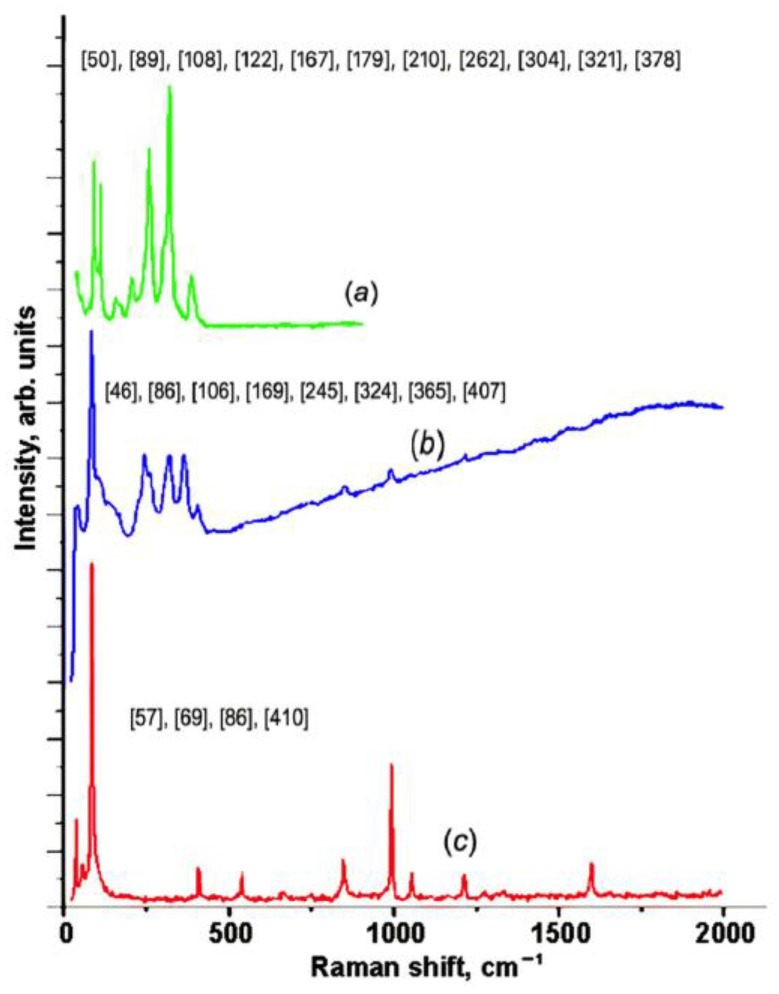
Raman spectra for the matrix crystal (**a**); partially intercalated (**b**); and In_1.2_Ga_0.8_S_3_·0.5(NH_2_-C_5_H_4_N) intercalation compound (**c**).

Figure 7a–c shows the Raman spectra of the pristine and partially intercalated In_1.2_Ga_0.8_S_3_ crystals, as well as the completely intercalated In_1.2_Ga_0.8_S_3_·0.5(p-AP) compound, respectively. As expected, all Raman peaks of the matrix crystal are fixed in the range of 50–400 cm^−1^ where ten peaks are clearly distinguished, which is typical for ordinary inorganic compounds. The spectrum of the intercalation compound (Figure 7c) can be divided into two parts.

The low-frequency region below 400 cm^−1^ characterizes the matrix crystal, whereas the high-frequency region represents the p-AP molecule.

The simplified low-frequency region of the spectrum displays only three peaks at 57, 69, and 86 cm^−1^. This fact confirms that the structure of the matrix crystal is significantly transformed due to the intercalation process. Figure 7b displays the Raman spectra for the partially intercalated matrix crystals. The partial intercalation of the p-AP molecules is confirmed by the weakening of the p-AP molecules’ peak intensities. In this case, one can see that the low-frequency region of the spectrum is quite different from the same regions in the other two spectra.

### 4.5. Intercalation of the p-Ethylenediamine into the In_1.2_Ga_0.8_S_3_

**Figure 8:**
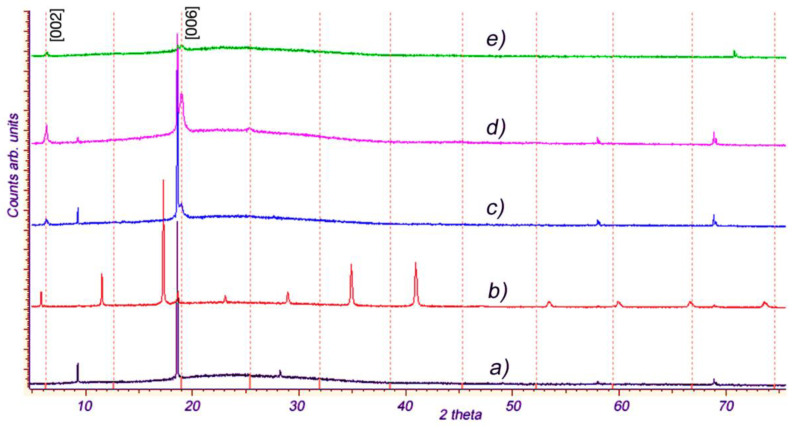
(**a**) XRD patterns for the pristine orthorhombic In_1.2_Ga_0.8_S_3_ crystal, (**b**) In_1.2_Ga_0.8_S_3_·0.5(p-AP) compound, and (**c**–**e**) p-EDA intercalated In_1.2_Ga_0.8_S_3_·0.5(p-AP).

The intercalation of p-AP molecules into In_1.2_Ga_0.8_S_3_ single crystals under given conditions occurs naturally. However, our attempts to synthesize analogous intercalation compounds with some related organic molecules, such as 2-AP, 3-AP, p-phenylenediamine, pyrazine, piperazine, and p-ethylenediamine, were unsuccessful, which means that the intercalation process in orthorhombic crystal In_1.2_Ga_0.8_S_3_ is highly selective. We studied the interaction of the same molecules with the intercalated compound In_1.2_Ga_0.8_S_3_·0.5(p-AP). The purpose of these experiments was to replace the p-AP molecule with p-phenylenediamine, pyrazine, and p-ethylenediamine molecules. Experiments with p-phenylenediamine and pyrazine molecules were unsuccessful, while substitutions with p-ethylenediamine (NH_2_-CH_2_-CH_2_-NH_2_, hereafter coined p-EDA) molecules were accompanied by significant changes. Figure 8 represents the five XRD patterns for the pristine orthorhombic In_1.2_Ga_0.8_S_3_ crystal (*a*), In_1.2_Ga_0.8_S_3_·0.5(p-AP) intercalation compound (*b*), and various samples, respectively obtained by the interaction of p-EDA molecules with In_1.2_Ga_0.8_S_3_·0.5(p-AP) crystals (*c,d,e*). By comparing these diffraction patterns, one can say that the interaction of p-EDA with In_1.2_Ga_0.8_S_3_·0.5(p-AP) crystals is accompanied by significant structural changes in the latter one.

As can be seen, there is no diffraction line in the In_1.2_Ga_0.8_S_3_·0.5(p-AP) diffractogram (Figure 8b) that matches with patterns Figure 8c–e. This confirms the complete extraction of p-AP molecules from the In_1.2_Ga_0.8_S_3_·0.5(p-AP) crystals. Moreover, the appearance of typical diffraction lines of the orthorhombic In_1.2_Ga_0.8_S_3_ in the XRD patterns (Figure 8c,d) means that the In_1.2_Ga_0.8_S_3_ matrix crystals were partially recovered. The detection of new diffraction peaks in Figure 8c,d confirms that the p-EDA molecules replaced the p-AP molecules, and a new intercalation compound with p-EDA, namely In_1.2_Ga_0.8_S_3_·0.5(p-EDA) was formed. A weak absolute value and relatively large width of the new peaks in the patterns given in Figure 8c–e indicate the possible exfoliation of the matrix crystal during the intercalation of p-EDA molecules into the layers. The unit cell parameter *c* of the crystals intercalated with p-EDA, calculated from the diffraction patterns (Figure 8c–e) using the newly appeared peaks (2θ = 6.4° and 19.1°), is ~13.975 Å. Compared with In_1.2_Ga_0.8_S_3_·0.5(p-AP), *c* was reduced by ~1.9 Å. This change is quite expected since the size of the p-EDA molecule was relatively smaller than that of p-AP.

### 4.6. DFT Investigations of the Vibrational Properties

**Figure 9:**
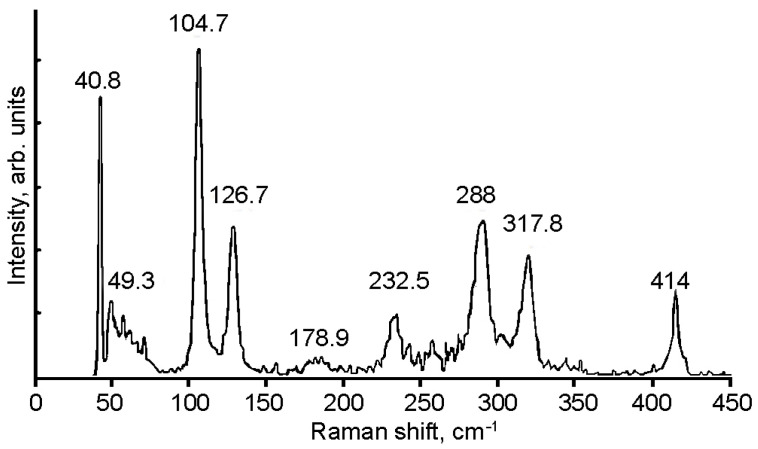
Raman spectrum of the GaInS_3_ compound.

There are ten atoms in the primitive cell of GaInS_3_ (Pearson’s card number 1,803,335 and JCPDS one 04-010-1075 [51]) and therefore, the phonon band structure has thirty normal phonon modes. The group-theoretical analysis produced the following phonon modes: Γ = 10A_1_ + 5A_2_ + 5B_1_ + 10B_2_, acoustic modes Γ_ac._ = A_1_ + B_1_ + B_2,_ and optical modes Γ_op._ = 9A_1_ + 5A_2_ + 4B_1_ + 9B_2_. All the latest modes are Raman active. A_1_, B_1,_ and B_2_ modes are also active in infrared reflection and therefore exhibit longitudinal-transverse optical splitting (LO-TO). Analysis of the displacement vector of atoms shows that, in A_2_ and B_1_ modes, the displacement of atoms occurs mainly along the crystallographic *x*-axes. Oscillations of the A_1_ and B_2_ symmetry modes occur mainly along the *x* and *y* crystallographic axes. Figure 9 shows the Raman spectra of GaInS_3_. Nine of twenty-seven Raman-active modes were detected from ab initio calculations of the phonon band structure (Table 1).

**Figure 10:**
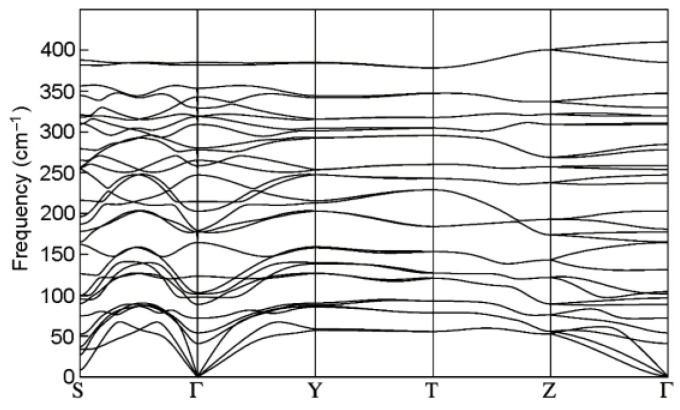
The phonon band dispersion of the GaInS_3_ compound.

**Figure 11:**
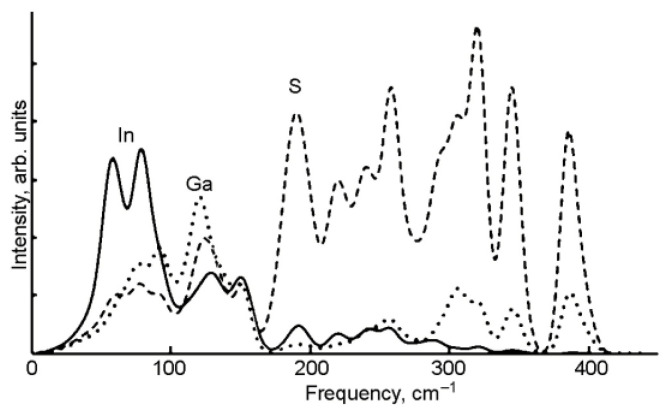
The atom projected phonon DOS of GaInS_3_.

The phonon mode dispersions of the GaInS_3_ along the symmetric lines of the Brillouin zone and atom-projected phonon density of states (PHDOS) are shown in Figure 10. As can be seen, the GaInS_3_ is dynamically stable, and the phonon band structure shows no imaginary modes. As can be seen from Figure 11, the first region from 0 to 164 cm^−1^ with a peak located at 81 cm^−1^ is dominated by the shift of heavy indium and gallium atoms and includes the acoustic and low-frequency optical modes. The second high-frequency region from 164 to 415 cm^−1^ results mainly from the shift of sulfur atoms in the unit cell, but with a small contribution of the gallium and indium atoms. The most intense peak of the Raman spectrum at 104.68 cm^−1^ corresponds to the A_1_ mode, in the vibrations of which all three Ga, In, and S atoms participate with almost the same contribution.

### 4.7. DFT Investigation of the Electronic Properties and Charge Density Topology

The structure was optimized by retaining the symmetry of the space group Cmc2_1_ (number 36, setting *Bb*2_1_*m*). In addition, the calculated cell parameters 18.86 × 6.168 × 3.846 Å are in excellent agreement with the experimental ones (19.06 × 6.194 × 3.811 Å) and recently calculated ones [54]. As expected, the van der Waals interaction energy is weak as it represents 6.6% of the total energy per unit cell.

**Figure 12:**
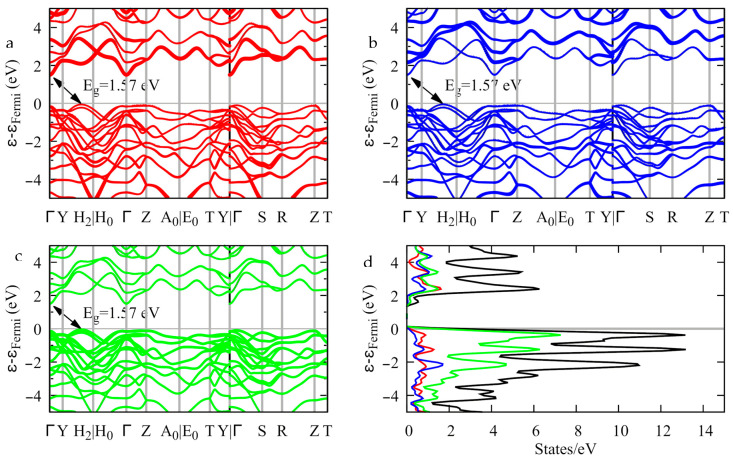
Band structure (**a**–**c**) and density of states (**d**) of InGaS_3_. The thickness of the bands is proportional to the contribution of the indium (**a**), gallium (**b**), and sulfur (**c**) orbitals.

The electronic band structure provides evidence of an indirect bandgap 1.57 eV wide. The top of the valence band is located between the Y and the H_2_ k-points, and the bottom of the conduction band is at Γ (Figure 12). As to the contributions of the atoms to the band edges, Figure 12 shows that the top of the valence band is mostly contributed by the sulfur atomic orbitals (Figure 12c), whereas the bottom of the conduction band is mostly contributed by the indium atomic orbitals (Figure 12a). Gallium contributes to both band edges to a lesser extent (Figure 12b).

**Figure 13:**
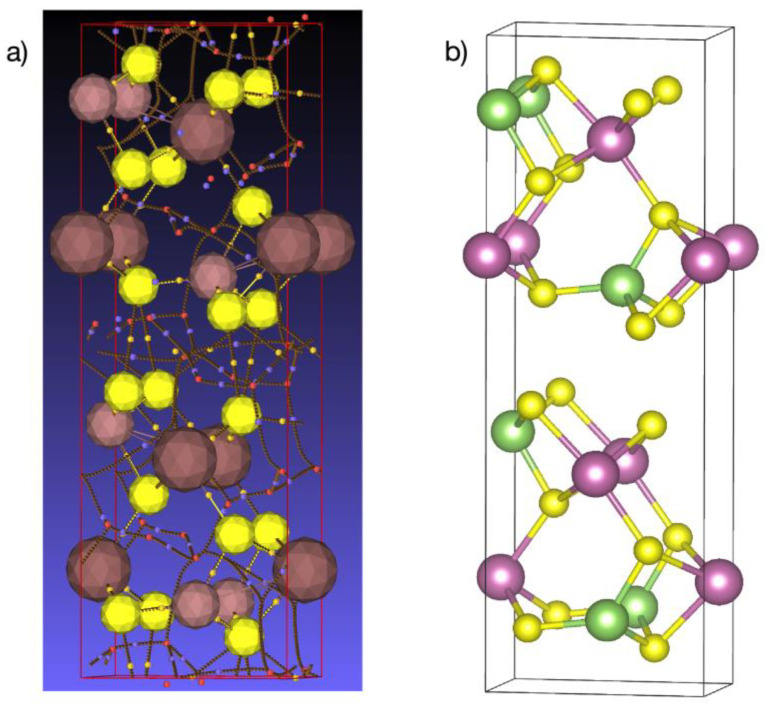
(**a**) Network of bond critical points (small yellow spheres), ring critical points (small red spheres), and cage critical points (small blue spheres). Sulfur atoms: large yellow spheres; gallium atoms: small brown spheres; and indium atoms: large brown spheres. (**b**) Corresponding atomic positions in the crystal structure. Sulfur in yellow, gallium in green, and indium in pink. View along the {001} direction; the gap between the layers is along the {100} direction.

The Bader topological analysis of the electron density features nine bond critical points (BCP) in the crystal structure. The bond critical points are gathered in Table S1 (see Supplemental Information) with some characteristic properties and are depicted in Figure 13b.

**Figure 14:**
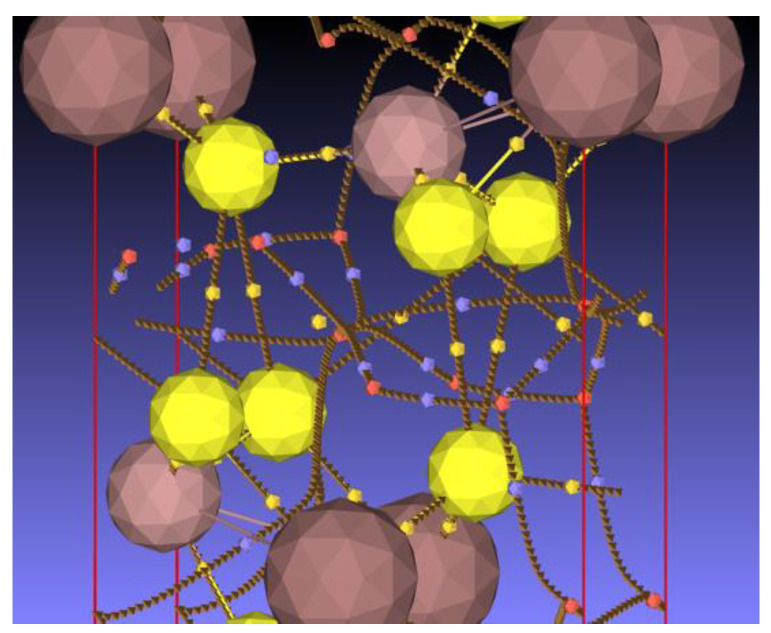
Focus on the bond, ring, and cage critical points and bond paths in the interlayer region of the structure. For the color legend, see the caption of Figure 13.

The electron density at the bond critical points located in the interlayer region is about one order of magnitude smaller than that between the other BCPs. These interlayer bond paths connect two sulfur atoms. A focus on this region is presented in Figure 14. The BCPs (small yellow spheres) are clearly located midway between the sulfur atoms, which is further confirmed by the d_1_/d_2_ ratio close to 1 (Table S1). By contrast, the BCPs located on the bond path connecting sulfur to gallium or sulfur to indium are more closely located to the metal, evidencing a larger basin volume for sulfur.

Both the electron density and the electron density Laplacian are small in the interlayer region compared to those of the BCPs located at the other bond paths (In-S and Ga-S). The positive value of the Laplacian shows that the electrons tend to escape from this region, exerting pressure on the surrounding electrons. Furthermore, the total energy density (H = G + V, see Table S1) and the bond degree H/ρ [55] are positive at these points in contrast to those at the Ga-S and In-S BCPs, which highlights the weak bonding between the layers. Starting from the relation H = G + V, the bond degree can be expressed with respect to the ratio V/G, which exhibits the competition between the potential and kinetic energy densities for bond formation, and to G/ρ which has been identified as the resistance of the bond electron cloud to deformation (reciprocal of the bond polarizability) [56]. The corresponding relation reads:

(1) $$\frac{H}{\rho }=\frac{G}{\rho }\left(1+\frac{V}{G}\right)$$

**Figure 15:**
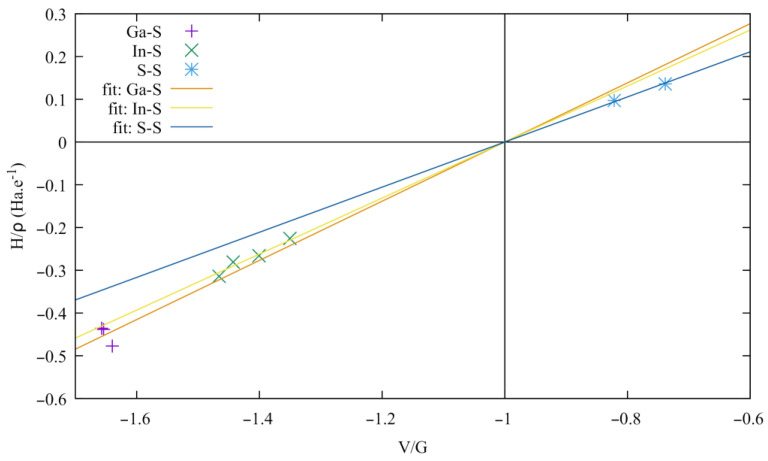
Bond degree versus V/G ratio at the bond critical point of Ga-S, In-S, and S-S bonding.

Realizing that G/ρ is a constant for a given bond, Equation (1) corresponds to an affine relation in which a line passes through the point (–1, 0). The bonding characteristics are plotted in Figure 15. Three distinct types of bonding can be seen, grouped as the S-S, In-S, and Ga-S bondings, corresponding to a closed-shell interaction for S-S and a shared-shell one for both In-S and Ga-S. The S-S bondings share the smallest G/ρ, thus exhibiting the largest polarizable bonding interaction, which can also be correlated to the largest bonding ellipticity ε = λ_1_/λ_2_ − 1 (Table S1).

Overall, the topological analysis shows that the bonding interaction between the layers is weak and involves sulfur atoms through a closed-shell interaction. Hence, upon the intercalation of organic molecules, the layers can be easily separated from each other to accommodate the molecules.

## 5. Conclusions

An intercalated In_1.2_Ga_0.8_S_3_·0.5(p-AP) compound was synthesized at 200 °C by the direct interaction of In_1.2_Ga_0.8_S_3_ matrix crystals with p-AP molecules. The lattice parameters of the triclinic cell were determined by XRD and a model structure of the In_1.2_Ga_0.8_S_3_·0.5(p-AP) intercalate was proposed. The temperature characteristics of deintercalation were determined by high-temperature XRD. A comparison of the Raman spectra of the matrix crystals and their intercalates revealed strong changes in the region of 0–400 cm^−1^, which are responsible for In-S and Ga-S bonds. In addition, the p-AP molecules in the In_1.2_Ga_0.8_S_3_·0.5(p-AP) intercalate were replaced by EDA molecules by direct interaction at 200 °C. When the latter crystals were kept at 200 °C for a few days, gradual amorphization occurred and the crystals exfoliated into elementary layers.

The dynamical properties of the stoichiometric GaInS_3_ were calculated by the DFPT method using GGA exchange-correlation potentials. Nine Raman active modes were identified from point group symmetry analysis. The phonon frequencies obtained by the ab initio lattice dynamics agreed well with the measured Raman modes. Additionally, the topological analysis of the charge density confirmed the weak interactions between the crystal layers involving sulfur atoms. The layers can thus be easily separated to accommodate organic molecules upon intercalation.

## Figures and Tables

**Table 1:** Calculated ω_theo_ and experimentally measured ω_exp_ eigenfrequencies of GaInS_3_. All frequencies are given in (cm^−1^). LO-TO splitting is indicated by a slash.

Mode							
A_1_(R, IR)		A_2_(R)		B_1_(R, IR)		B_2_(R, IR)	
ω_theo_|ω_exp_							
71.84		40.9	40.8	96.37		53.94	49.3
103.18	104.7	88.75		165.39		101.55	
123.15	126.7	177.15		254.04		164.38	
179.1	178.9	258.5		310.54		202.84	
214.74		309.1				240.24/247.24	232.5
265.14						283.18/278.18	288.0
318.71	317.8					319.55	
342.75						329.72	
382.2						407.2/385.2	414.0

## Data Availability

Data are available upon request to the corresponding authors.

## Supplementary Materials

The following supporting information can be downloaded at: https://www.mdpi.com/article/10.3390/ma16062368/s1, Table S1: Bond critical points (BCP) with properties: atom ends connected by the BCP, interatomic distance *d*, the ratio of the distances between the BCP and the atom ends d_1_/d_2_, angle θ between the BCP and the atom ends (BCP being the apex), electron density Laplacian eigenvalues λ_i_, (i = 1, 2, 3), ellipticity λ_1_/λ_2_-1, electron density Laplacian ∇^2^ρ, the electron density in ρ in Bohr^−3^, kinetic energy density G in Ha.Bohr^–3^, potential energy density V in Ha.Bohr^−3^, bond degree H/ρ in Ha.e^−1^, potential to kinetic energy density ratio V/G, and kinetic energy per electron G/ρ in Ha.e^−1^.
