# Supplementary material for: Intercalation of p-Aminopyridine and p-Ethylenediamine Molecules into Orthorhombic In1.2Ga0.8S3 Single Crystals
Source: Materials (Basel). 2023 Mar 15;16(6):2368. doi: 10.3390/ma16062368 (PMC10053688; doi:10.3390/ma16062368)
Supplement: Supplementary file 1 [file materials-16-02368-s001.zip › materials-2211467-supplementary.pdf]

materials

Article

Supplemental data to "Intercalation of p-Aminopyridine and p-Ethylenediamine Molecules into Orthorhombic $In_{1.2}Ga_{0.8}S_3$ Single Crystals"

Aysel B. Rahimli [1], Imamaddin R. Amiraslanov [1,2,*], Zakir A. Jahangirli [1,2], Naila H. Aliyeva [3], Pascal Boulet [4], Marie-Christine Record [5] and Ziya S. Aliev [2,*]

[1] Institute of Physics, Ministry of Science and Education of Azerbaijan, AZ1143 Baku, Azerbaijan
[2] Nanoresearch Laboratory, Baku State University, AZ1148 Baku, Azerbaijan
[3] Chemical Technologies Department, Faculty of Metallurgy and Materials Science, Azerbaijan Technical University, AZ1073 Baku, Azerbaijan
[4] CNRS MADIREL Laboratory, Faculty of Sciences, Aix-Marseille University, 13013 Marseille, France
[5] CNRS IM2NP Laboratory, Faculty of Sciences, Aix-Marseille University, 13013 Marseille, France
* Correspondence: iamiraslan@gmail.com (I.R.A.); ziyasaliev@gmail.com (Z.S.A.)

Table S1. Bond critical points (BCP) with the following properties: atom ends connected by the BCP, interatomic distance *d*, the ratio of the distances between the BCP and the atom ends d_1/d_2, angle θ between the BCP and the atom ends (BCP being the apex), electron density Laplacian eigenvalues λ_i,(i = 1, 2, 3), ellipticity λ_1/λ_2-1, electron density Laplacian $\nabla^2\rho$, the electron density in ρ in Bohr^{-3}, the kinetic energy density G in Ha.Bohr^{-3}, the potential energy density V in Ha.Bohr^{-3}, the bond degree H/ρ in Ha.e^{-1}, potential to kinetic energy density ratio |V|/G, and the and kinetic energy per electron G/ρ in Ha.e^{-1}.

BCP	Atoms	d (Å)	d_1/d_2	θ (°)	$\lambda_1 \times 10^2$	$\lambda_2 \times 10^2$	$\lambda_3 \times 10^2$	$\lambda_1/\lambda_2 - 1 \times 10^2$	$\nabla^2\rho \times 10^2$	$\rho \times 10^2$	$G \times 10^2$	$V \times 10^2$	H/ρ	V/G	G/ρ
b1	S-S	3.873	0.995	179.68	−0.226	−0.206	1.71	9.42	1.28	0.489	0.254	−0.187	0.136	−0.738	0.520
b2	S-S	3.589	0.995	178.71	−0.468	−0.430	3.00	8.76	2.11	0.820	0.447	−0.367	0.0971	−0.822	0.545
b3	In-S	2.658	0.903	179.85	−4.64	−4.38	16.3	5.83	7.31	5.17	3.28	−4.73	−0.281	−0.144	0.634
b4	In-S	2.708	0.902	178.50	−3.88	−3.77	15.2	2.97	7.59	4.53	2.92	−3.94	−0.226	−1.35	0.644
b5	In-S	2.628	0.912	179.85	−4.61	−4.57	17.4	0.984	8.23	5.17	3.43	−481	−0.266	−1.40	0.664
b6	In-S	2.578	0.912	177.94	−5.46	−5.37	19.3	1.60	8.47	5.88	3.96	−5.81	−0.314	−1.47	0.674
b7	Ga-S	2.343	0.809	178.34	−7.56	−7.26	21.6	4.17	6.83	7.52	4.98	−8.25	−0.436	−1.66	0.663
b8	Ga-S	2.343	0.809	178.29	−7.67	−7.32	22.1	4.84	7.07	7.61	5.11	−8.44	−0.439	−1.65	0.670
b9	Ga-S	2.256	0.777	178.41	−8.91	−8.66	27.0	2.84	9.43	8.77	6.54	−10.7	−0.477	−1.64	0.746
